# Inclusive leadership can improve nurses’ psychological ownership and reduce their turnover intention under the normalization of COVID-19 prevention

**DOI:** 10.3389/fpsyg.2022.1100172

**Published:** 2023-01-09

**Authors:** Dongyu Zeng, Baoxiang Wang, Weiju Chen

**Affiliations:** School of Nursing, Jinan University, Guangzhou, China

**Keywords:** inclusive leadership, psychological ownership, turnover intention, COVID-19, normalization

## Abstract

**Introduction:**

The COVID-19 pandemic continues to impact people’s lives and professions worldwide. Chinese nurses face immense work pressure under the normalization of COVID-19 prevention and control, resulting in greater turnover intention. It is, therefore, crucial to study the mechanisms that influence the turnover intention of nurses in this situation.

**Objective:**

Many studies have examined the impact of leadership style on nurses’ turnover intention; however, few researchers have investigated this influence during the COVID-19 pandemic. Based on the leader-member exchange theory, this study empirically studied the effect of inclusive leadership on turnover intention of nurses under the normalization of COVID-19 prevention and control in China, while assessing the mediating role of psychological ownership.

**Design:**

Cross-sectional study with multi-center data.

**Participants:**

Two thousand, two hundred ninety-nine registered nurses from 17 hospitals in China were recruited from January to March, 2022, under the normalization of COVID-19 prevention and control in China.

**Methods:**

A demographic questionnaire and scales of inclusive leadership, psychological ownership, and turnover intention integrated into an online survey were sent to registered nurses of different hospitals. Maximum likelihood structural equation modeling (ML-SEM) was used to analyze data.

**Results:**

Independent variable inclusive leadership has a significant effect on the overall turnover intention of nurses, *p* < 0.001. The direct effect path coefficient from inclusive leadership to psychological ownership is significant, *p* < 0.001. The direct effect path coefficient from psychological ownership to turnover intention is significant, *p* < 0.001. The indirect effect path coefficient from inclusive leadership to turnover intention is significant, *p* < 0.001.

**Conclusion:**

Psychological anxiety, burnout, turnover intention, and even suicidal thoughts were the main symptoms of Chinese nurses under the normalization of COVID-19 prevention and control in China. The absence of a mechanism to counteract these negative conditions may ultimately lead to personal psychological distress for nurses and collapse of the healthcare system. Inclusive leadership can improve nurses’ psychological ownership level and reduce their turnover intention by treating them fairly, providing them with opportunities for self-development, paying attention to communication with them, and increasing their sense of belonging, self-efficacy, and self-identity.

## 1. Introduction

COVID-19 was declared a global public health emergency on 30 January, 2020. According to the COVID-19 Real-time Big Data report, as of 3 November, 2022, there were 630,452,398 confirmed cases and 6,590,206 deaths worldwide. The number of new confirmed cases continues to increase every day, led by the India, United States, and France. China continues to face the pressure of prevention and control from external import and internal rebound, and the situation of prevention and control of COVID-19 is still challenging (Wu et al., 2021). In 2020, nurses constituted approximately 70% of the medical staff supporting the fight against COVID-19 in the Hubei Province. The majority of nurses have been serving at the forefront of clinical practice and constitute the main force in the response to this pandemic over the last 2 years, especially in the context of normalization of COVID-19 prevention in China. Normalization of COVID-19 prevention in China implies the country has implemented epidemic prevention measures, such as wearing masks, maintaining social distancing, checking body temperature, and conducting regular nucleic acid tests into daily production and daily life, and the Chinese people have basically returned to normal life as result of these measures. However, during normalization of prevention, nurses in the front line are under tremendous work pressure, which significantly impacts their lives, manifesting as psychological anxiety (Park et al., 2021), job burnout (Schug et al., 2022), increased turnover intention (Cole et al., 2021; Khattak et al., 2021; Labrague and de los Santos, 2021), and even suicide. A study in Wuhan, China, investigated nurses in the ‘post-epidemic era’ and identified 73% of them had high turnover intention (Li et al., 2021). As the largest group of healthcare providers (Drennan and Ross, 2019), nurses play an irreplaceable role in healthcare. The loss of nurses will have a serious adverse impact on the normal operation of the hospital, and will significantly hinder the development of the entire healthcare system (Simon, 2014). Therefore, in the context of the COVID-19 pandemic, it is crucial to clarify the factors causing stress to nurses and remedies for high turnover intention.

The uncertainty about the end of the pandemic, the need to wear cumbersome personal protective equipment for extended periods, the fear of infection (Lai et al., 2020; Razu et al., 2021), and the impatience of patients (Sovold et al., 2021) were the main factors behind the psychological distress and high turnover intention of front-line nurses during the pandemic. Leadership, especially inclusive leadership, has been shown to be effective in helping nurses out of psychological distress and reduce their turnover intention. Inclusive leadership is a leadership style that shows openness, effectiveness, and accessibility in the process of interaction with subordinates (Carmeli et al., 2010). Inclusive leadership facilitates a relaxed state of mind, improves psychological safety (Lee and Dahinten, 2021), and reduces psychological pain of nurses (Ahmed et al., 2020). Psychological ownership refers to a certain kind of psychological state of people, denoted by their emotions and cognition toward their organization, including the sense of belonging, self-efficacy, and self-identity. When nurses’ sense of belonging, self-efficacy, and self-identity improve because of inclusive leadership, their sense of responsibility is automatically triggered, increasing their intention to give more time and energy to the organization. Consequently, their turnover intention decreases. Hence, psychological ownership—already proven to have a mediating effect—can improve nurses’ job satisfaction and negatively impact their turnover intention (Jing and Yan, 2022), so as to promote the stable development of hospitals (Schirle and Dietrich, 2020).

Most of the previous studies on nurses’ turnover intentions focussed on job satisfaction, burnout, work pressure, performance, etc., (Lee and Kim, 2020; Guo et al., 2021); little attention was paid to the influence of leadership style on nurses’ turnover intention. During negative events, specifically, traumatic events, disasters, or public health emergencies, there is even less research on the influence of leadership style on nurse turnover intention. Few studies have explored the impact of leadership style on turnover intention among nurses during the COVID-19 pandemic; this study intends to fill that research gap. This study focusses on a relational leadership style called inclusive leadership to explore the influence mechanism of inclusive leadership on nurses’ turnover intention under the COVID-19 pandemic. Variables: different hospital settings, age, marital status, educational background, work relationship, inclusive leadership, psychological ownership, and turnover intention were selected to verify the negative predictive effects of inclusive leadership on turnover intention during the pandemic, and to test the mediating effect of psychological ownership between inclusive leadership and turnover intention of nurses. The findings will make theoretical and empirical contributions to inclusive leadership theory research and provide further implications for nursing managers responding to human resource management of staff during the pandemic.

## 2. Materials and methods

### 2.1. Study design and participants

This is a cross-sectional study. A demographic questionnaire and scales of inclusive leadership, psychological ownership, and turnover intention were integrated into an online survey. A weblink and QR code were created and distributed through WeChat to the directors of nursing departments of 17 hospitals in the Guangzhou, Shenzhen, Huizhou, Maoming, Zhanjiang, and Guangdong Province, China, from January to March, 2022. Issues such as disclosure statement and informed consent–these were mentioned on the front page of the online survey–were duly explained to all directors by authors. The directors sent out the weblink and QR code to the registered nurses working in various departments through WeChat, who participated voluntarily. The disclosure statement clarified that only registered nurses should participate. We recommended those registered nurses to not participate who were on leave or at work in the administrative department, supply room, logistics, or other posts that do not provide direct care for patients; the responses of such participants were discarded during data screening.

### 2.2. Measures

Based on literature review and analysis, the items of demographic information were prepared and the scales of inclusive leadership, psychological ownership, and turnover intention were adopted from previous studies. These scales were all mature and with high reliability and validity. The items of the Inclusive Leadership Scale, Psychological Ownership Scale, and Turnover Intention Scale were assessed on a 5-point Likert scale ranging from 1 = strongly disagree to 5 = strongly agree.

The demographic questionnaire includes 19 items, including gender, age, educational background, working years, professional title, position, hospital grade, department, marital status, children, number of night shifts per week, working hours per week, monthly income, etc.

The Inclusive Leadership Scale compiled by Carmeli et al. (2010) was adopted in our study. It contains nine items in three dimensions: openness, accessibility, and usability. Sample items include, ‘The manager is open to hearing new ideas’ and ‘The manager encourages me to access him/her regarding emerging issues’. It has Cronbach’s alpha of 0.953, showing good internal consistency.

This study adopted the Psychological Ownership Scale (seven items) developed by Van Dyne and Pierce (2004). It consists of seven items that use words like “MY”, or “OUR” to ask participants to describe their “sense of ownership”. For example: “This is MY organization.”, “I sense that this organization is OUR company.”, “It is hard for me to think about this organization as MINE (reversed)”. This scale has been widely recognized and praised by the academic community since its publication. Presently, most domestic scholars use this scale for research, and Cronbach’s alpha shows excellent internal consistency reliability of over 0.87.

The Turnover Intention Scale (six items) used for this survey was developed by Michaels and Spector (1982) in 1982, and translated into Chinese and revised by Li and Li (2003). According to the correlation between items, Items 1 and 6 represent the possibility of quitting the current job. Items 2 and 3 denote the possibility of seeking other jobs. Items 4 and 5 refer to the possibility of obtaining other jobs. The reliability coefficient of this scale is 0.773, which has a credible internal consistency and meets the statistical requirements.

### 2.3. Data analysis

The data were analyzed using SPSS 26.0 and Amos 21.0. Maximum likelihood structural equation modeling (ML-SEM) was used as it enables researchers to modify the model based on the results of data analysis. Therefore, modification indices were also computed, and correlations were established between two residuals with a higher modification index. The established correlation between two residuals explained the possible interaction between two different observed variables (Wu, 2007; Qiu, 2019).

## 3. Results

### 3.1. Sample

There were 2,299 complete responses. The data were analyzed using IBM SPSS Statistics Pack 26.0 software. Table 1 presents the demographic characteristics of the sample including grade and professional character of hospital, work department, educational and vocational background, work status, the extent of family support for current job, etc. Approximately 72.1% (*n* = 1,657) of the participants were nurses from Grade 3 general hospitals; 47.6% (*n* = 1,095) were from hospitals that have received COVID-19 patients; 77.7% (*n* = 1787) were performing clinical nursing work; 79.6% responded that they got along well with colleagues; 67.1% responded that they got along well with their immediate leaders; 72.6% (*n* = 1,669) stated they received family support for their current jobs; and 74.2% (*n* = 1707) were working in their own cities.

**Table 1:** Demographic characteristics of participants.

Variable	No. (%)	Variable	No. (%)
Grade of hospital		Labor relations	
Grade 1	177 (7.7)	Official personnel establishment	796 (34.6)
Grade 2	465 (20.2)	Engagement	232 (10.1)
Grade 3	1,657 (72.1)	Contract labor	1,271 (55.3)
Professional character of hospital		Marriage	
Specialized hospital	114 (5.0)	Married	1,571 (68.3)
General hospital	2,185 (95.0)	Unmarried	728 (31.7)
Designated hospitals for COVID-19 patients		Children	
Yes	868 (37.8)	0	878 (38.2)
No	1,431 (62.2)	1	711 (30.9)
Received COVID-19 patients		2	686 (29.8)
Yes	1,095 (47.6)	Above 2	24 (1.0)
No	1,204 (52.4)	Years of working	
Work department		<1	103 (4.5)
Internal medicine	640 (27.8)	1–5	560 (24.4)
Surgery	445 (19.4)	6–10	688 (29.9)
Gynecology	76 (3.3)	11–15	400 (17.4)
Obstetrics	134 (5.8)	≥16	548 (23.8)
Pediatrics	108 (4.7)	No. of night shifts per month	
Emergency department	227 (9.9)	0–3	885 (38.5)
Intensive care unit	150 (6.5)	4–6	1,237 (53.8)
Fever clinic	92 (4.0)	≥7	177 (7.7)
Isolation ward	20 (0.9)	Working hours per week	
Other	407 (17.7)	≤40	577 (25.1)
Gender		41–48	1,104 (48.0)
Male	62 (2.7)	>48	618 (26.9)
Female	2,237 (97.3)	After-tax income	
Age		≤5,000	573 (24.9)
≤25	421 (18.3)	6,000–9,000	1,348 (58.6)
26–35	1,178 (51.2)	≥10,000	378 (16.4)
36–45	520 (22.6)	Getting along with colleagues	
≥46	180 (7.8)	Good	1831 (79.6)
Education background		General	464 (20.2)
Polytechnic school	76 (3.3)	Bad	4 (0.2)
Junior college	930 (40.5)	Getting along with immediate leaders	
Undergraduate	1,282 (55.8)	Good	1,542 (67.1)
Graduate and above	11 (0.5)	General	726 (31.6)
Professional title		Bad	31 (1.3)
Nurse	549 (23.9)	Family support for current job	
Senior nurse	936 (40.7)	Support	1,669 (72.6)
Supervisor nurse	668 (29.1)	Not support, not object	602 (26.2)
Co-chief superintendent nurse and above	146 (6.4)	Object	28 (1.2)
Position		Census register	
Clinical nurse	1787 (77.7)	Local city	1707 (74.2)
Group leader	297 (12.9)	Other cities of local province	337 (14.7)
Head nurse	198 (8.6)	Other provinces	255 (11.1)
Director or deputy director of nursing department	17 (0.7)		

### 3.2. Responses to the three questionnaires

Table 2 presents how respondents rated each item in the questionnaires on inclusive leadership, psychological ownership, and turnover intention.

**Table 2:** Responses to questionnaires.

Group	Inclusive leadership			Psychological ownership			Turnover intention		
	*x* ± *s*	*t*/*F*	*P*	*x* ± *s*	*t*/*F*	*P*	*x* ± *s*	*t*/*F*	*P*
Grade of hospital									
Grade 1	3.97 ± 0.807	8.687	0.000***	3.66 ± 0.600	5.167	0.006**	2.24 ± 0.632	7.701	0.000***
Grade 2	3.86 ± 0.735			3.52 ± 0.614			2.44 ± 0.644		
Grade 3	4.03 ± 0.758			3.61 ± 0.620			2.45 ± 0.654		
Professional character of hospital									
Specialized hospital	3.91 ± 0.777	1.258	0.262	3.56 ± 0.618	0.330	0.566	2.50 ± 0.616	1.543	0.214
General hospital	3.99 ± 0.759			3.60 ± 0.619			2.43 ± 0.654		
Designated hospitals for COVID-19 patients									
Yes	4.03 ± 0.773	4.055	0.044*	3.61 ± 0.635	0.847	0.358	2.45 ± 0.645	1.307	0.253
No	3.97 ± 0.751			3.59 ± 0.608			2.42 ± 0.656		
Received COVID-19 patients									
Yes	3.99 ± 0.762	0.046	0.830	3.58 ± 0.638	1.696	0.193	2.46 ± 0.637	5.164	0.023*
No	3.99 ± 0.759			3.61 ± 0.600			2.40 ± 0.664		
Work department									
Internal medicine	4.00 ± 0.771	3.801	0.000***	3.57 ± 0.631	3.479	0.000***	2.48 ± 0.648	4.516	0.000***
Surgery	4.08 ± 0.803			3.62 ± 0.639			2.43 ± 0.655		
Gynecology	3.98 ± 0.780			3.55 ± 0.77			2.40 ± 0.725		
Obstetrics	3.91 ± 0.762			3.64 ± 0.557			2.48 ± 0.628		
Pediatrics	3.91 ± 0.713			3.66 ± 0.600			2.39 ± 0.643		
Emergency department	3.86 ± 0.734			3.48 ± 0.638			2.41 ± 0.646		
Intensive care unit	3.79 ± 0.740			3.46 ± 0.599			2.63 ± 0.679		
Fever clinic	4.18 ± 0.708			3.66 ± 0.587			2.38 ± 0.664		
Isolation ward	3.84 ± 0.937			3.50 ± 0.621			2.53 ± 0.604		
Other	4.03 ± 0.702			3.70 ± 0.549			2.29 ± 0.620		
Gender									
Male	3.84 ± 1.017	1.364	0.247	3.43 ± 0.840	2.507	0.118	2.55 ± 0.755	2.235	0.135
Female	3.99 ± 0.752			3.60 ± 0.611			2.43 ± 0.649		
Age									
≤25	3.88 ± 0.776	10.771	0.000***	3.44 ± 0.624	58.847	0.000***	2.59 ± 0.610	121.464	0.000***
26–35	3.95 ± 0.771			3.52 ± 0.628			2.56 ± 0.622		
36–45	4.10 ± 0.729			3.77 ± 0.550			2.20 ± 0.612		
≥46	4.17 ± 0.670			3.93 ± 0.481			1.85 ± 0.537		
Education background									
Polytechnic school	3.82 ± 0.879	3.514	0.015*	3.52 ± 0.688	2.544	0.055	2.30 ± 0.721	1.252	0.289
Junior college	3.95 ± 0.756			3.57 ± 0.618			2.43 ± 0.659		
Undergraduate	4.03 ± 0.755			3.62 ± 0.614			2.44 ± 0.644		
Graduate and above	4.18 ± 0.510			3.86 ± 0.461			2.58 ± 0.491		
Professional title									
Nurse	3.9 ± 0.760	22.465	0.000***	3.49 ± 0.613	59.718	0.000***	2.55 ± 0.632	87.940	0.000***
Senior nurse	3.92 ± 0.802			3.51 ± 0.649			2.56 ± 0.645		
Supervisor nurse	4.09 ± 0.696			3.72 ± 0.553			2.27 ± 0.604		
Co-chief superintendent nurse and above	4.32 ± 0.620			3.99 ± 0.438			1.88 ± 0.527		
Position									
Clinical nurse	3.93 ± 0.773	21.717	0.000***	3.53 ± 0.624	59.465	0.000***	2.50 ± 0.643	64.472	0.000***
Group leader	4.12 ± 0.715			3.70 ± 0.577			2.37 ± 0.638		
Head nurse	4.29 ± 0.618			3.98 ± 0.458			1.93 ± 0.538		
Director or deputy director of nursing department	4.22 ± 0.570			3.97 ± 0.316			2.13 ± 0.384		
Labor relations									
Official personnel establishment	4.08 ± 0.736	11.067	0.000***	3.76 ± 0.555	55.606	0.000***	2.17 ± 0.615	111.612	0.000***
Engagement	4.04 ± 0.719			3.67 ± 0.585			2.42 ± 0.622		
Contract labor	3.92 ± 0.776			3.48 ± 0.637			2.59 ± 0.628		
Marriage									
Married	4.05 ± 0.735	29.116	0.000***	3.68 ± 0.596	87.568	0.000***	2.35 ± 0.661	85.021	0.000***
Unmarried	3.87 ± 0.798			3.42 ± 0.631			2.60 ± 0.598		
Children									
0	3.89 ± 0.798	9.052	0.000***	3.44 ± 0.643	29.127	0.000***	2.59 ± 0.611	28.921	0.000***
1	4.03 ± 0.725			3.67 ± 0.580			2.32 ± 0.657		
2	4.07 ± 0.732			3.71 ± 0.582			2.34 ± 0.655		
Above 2	4.06 ± 0.779			3.62 ± 0.689			2.28 ± 0.786		
Years of working									
<1	4.11 ± 0.686	12.165	0.000***	3.65 ± 0.510	45.024	0.000***	2.38 ± 0.552	77.733	0.000***
1–5	3.86 ± 0.774			3.42 ± 0.640			2.62 ± 0.605		
6–10	3.91 ± 0.794			3.49 ± 0.650			2.58 ± 0.639		
11–15	4.08 ± 0.728			3.69 ± 0.549			2.42 ± 0.619		
≥16	4.13 ± 0.707			3.83 ± 0.528			2.05 ± 0.592		
No. of night shift per month									
0–3	4.13 ± 0.714	28.118	0.000***	3.75 ± 0.564	45.573	0.000***	2.22 ± 0.625	77.948	0.000***
4–6	3.92 ± 0.772			3.50 ± 0.632			2.57 ± 0.625		
≥7	3.78 ± 0.787			3.52 ± 0.635			2.49 ± 0.701		
Working hours per week									
≤40	4.12 ± 0.730	14.094	0.000***	3.68 ± 0.595	8.344	0.000***	2.31 ± 0.645	13.595	0.000***
41–48	3.98 ± 0.728			3.59 ± 0.599			2.45 ± 0.633		
>48	3.88 ± 0.824			3.53 ± 0.665			2.50 ± 0.679		
After-tax income									
≤5,000	3.85 ± 0.766	32.088	0.000***	3.48 ± 0.644	46.494	0.000***	2.54 ± 0.652	41.298	0.000***
6,000–9,000	3.98 ± 0.753			3.57 ± 0.604			2.46 ± 0.641		
≥10,000	4.25 ± 0.715			3.86 ± 0.556			2.16 ± 0.623		
Getting along with colleagues									
Good	4.13 ± 0.705	173.710	0.000***	3.70 ± 0.580	139.282	0.000***	2.34 ± 0.640	93.867	0.000***
General	3.44 ± 0.717			3.19 ± 0.598			2.78 ± 0.577		
Bad	3.64 ± 1.322			3.29 ± 0.841			2.25 ± 0.833		
Getting along with immediate leaders									
Good	4.28 ± 0.627	515.152	0.000***	3.78 ± 0.550	255.906	0.000***	2.28 ± 0.629	148.737	0.000***
General	3.45 ± 0.643			3.25 ± 0.578			2.71 ± 0.586		
Bad	2.43 ± 0.636			2.70 ± 0.581			3.15 ± 0.582		
Family support for current job									
Support	4.15 ± 0.691	132.763	0.000***	3.74 ± 0.564	169.630	0.000***	2.28 ± 0.630	196.729	0.000***
Not support, not object	3.59 ± 0.760			3.24 ± 0.590			2.81 ± 0.539		
Object	3.19 ± 1.022			2.94 ± 0.781			2.96 ± 0.648		
Census register									
Local city	4.02 ± 0.742	6.057	0.002**	3.64 ± 0.612	17.812	0.000***	2.37 ± 0.659	30.552	0.000***
Other cities of local province	3.86 ± 0.778			3.48 ± 0.60			2.58 ± 0.571		
Other provinces	3.96 ± 0.835			3.45 ± 0.646			2.62 ± 0.637		

Statistically significant values ^*^p < 0.05; ^**^p < 0.01; ^***^p < 0.001.

### 3.3. Assessment results of the measurement model

Confirmatory factor analysis was used to assess the psychometric features of the measurement model. The item reliability of a construct is determined by its factor loading. Table 3 shows that the factor loading ranged between 0.256 and 0.924. Most of the item values were higher than 0.70, with significant t-statistics (*p* < 0.001).

**Table 3:** Confirmatory factor analysis.

Indicators	Factor loadings	Mean	Standard deviation	*T* statistics	*P*-values
IL1 < - IL	0.844	3.94	0.875	/	/
IL2 < - IL	0.852	3.94	0.857	63.838	<0.001
IL3 < - IL	0.851	3.99	0.827	58.421	<0.001
IL4 < - IL	0.882	4.03	0.856	53.378	<0.001
IL5 < - IL	0.865	4.09	0.821	48.144	<0.001
IL6 < - IL	0.868	4.08	0.812	48.626	<0.001
IL7 < - IL	0.901	3.86	0.934	55.737	<0.001
IL8 < - IL	0.900	3.99	0.866	52.432	<0.001
IL9 < - IL	0.900	3.99	0.833	53.984	<0.001
PO1 < - PO	0.813	3.99	0.896	/	/
PO2 < - PO	0.853	4.00	0.902	46.256	<0.001
PO3 < - PO	0.894	3.71	1.005	51.145	<0.001
PO4 < - PO	0.814	3.60	1.075	43.487	<0.001
PO5 < - PO	0.862	3.97	0.884	44.981	<0.001
PO6 < - PO	0.786	3.49	1.008	40.177	<0.001
PO7 < - PO	0.759	3.58	0.948	48.037	<0.001
TI1 < - TI	0.924	2.37	0.97	/	/
TI2 < - TI	0.726	2.15	0.928	41.575	<0.001
TI3 < - TI	0.791	2.27	0.971	47.413	<0.001
TI4 < - TI	0.256	2.87	0.729	10.723	<0.001
TI5 < - TI	0.416	2.75	0.751	17.96	<0.001
TI6 < - TI	0.787	2.17	0.885	47.066	<0.001

IL, Inclusive Leadership; PO, Psychological Ownership; TI, Turnover Intention.

Model fit indices were used to examine how well the model fits the data. They include χ^2^, χ^2^/df (Degrees of Freedom), GFI (Goodness of Fit Index), AGFI (Adjust Goodness of Fit Index), NFI (Normed Fit Index), IFI (Incremental Fit Index), TLI (Tucker-Lewis Index), CFI (Comparative Fit Index) and RMSEA (Root Mean Square Error of Approximation). Model fit indices for each construct were calculated and showed as Table 4, which confirmed that each model fits the data very well.

**Table 4:** Model Fit Indices.

	χ2	χ2/df	GFI	AGFI	NFI	IFI	TLI	CFI	RMSEA
IL	17.613	3.523	0.998	0.985	0.999	0.999	0.996	0.999	0.033
PO	24.016	6.004	0.997	0.980	0.998	0.998	0.992	0.998	0.047
TI	8.897	2.224	0.999	0.993	0.999	0.999	0.997	0.999	0.023

IL, Inclusive Leadership; PO, Psychological Ownership; TI, Turnover Intention.

Composite reliability (CR) was used to determine how well its items measure a construct. The CR scores of all the constructs were higher than 0.8 (recommended value is 0.70), which indicates that all the constructs were of good internal consistency and reliability. Based on the Fornell and Larcker (1981) criterion, Average Variance Extracted (AVE) values were used to determine the convergent validity of the constructs. The AVE value of Turnover Intention was 0.483, which is close to 0.50 (recommended value is 0.70). It is sufficient to prove its convergent validity (Lam, 2012). The AVE values of the Inclusive Leadership and Psychological Ownership were higher than 0.50. The convergent validity of the constructs was confirmed.

Discriminant validity was used to show the extent of difference of each construct. We can see from Table 5 that the square root of AVE values was among diagonal, whereas correlation weights were below. All the correlation weights were lower than the AVE values. The discriminant validity was confirmed.

**Table 5:** Discriminant validity.

	IL	PO	TI
IL	0.874		
PO	0.744***	0.820	
TI	−0.509***	−0.683***	0.695

IL, Inclusive Leadership; PO, Psychological Ownership; TI, Turnover Intention.

Statistically significant value ^***^p < 0.001.

### 3.4. Assessment of the structural model

Structural equation model was assessed using Amos 21.0 to study the relationship between inclusive leadership, psychological ownership, and turnover intention, and whether psychological ownership acts as a mediator between inclusive leadership and turnover intention. We followed Wen’s method to examine both partial mediation and complete mediation (Wen and Ye, 2014) using the following specific steps: 1. Examine the significant impact of inclusive leadership on the turnover intention of nurses, 2. Examine the effect of inclusive leadership on nurses’ psychological ownership, 3. Additionally, inclusive leadership, psychological ownership, and turnover intention were incorporated into the structural equation. In the causal impact analysis, psychological ownership–as the outcome variable of inclusive leadership—was also the antecedent variable of nurses’ turnover intention.

**Figure 1:**
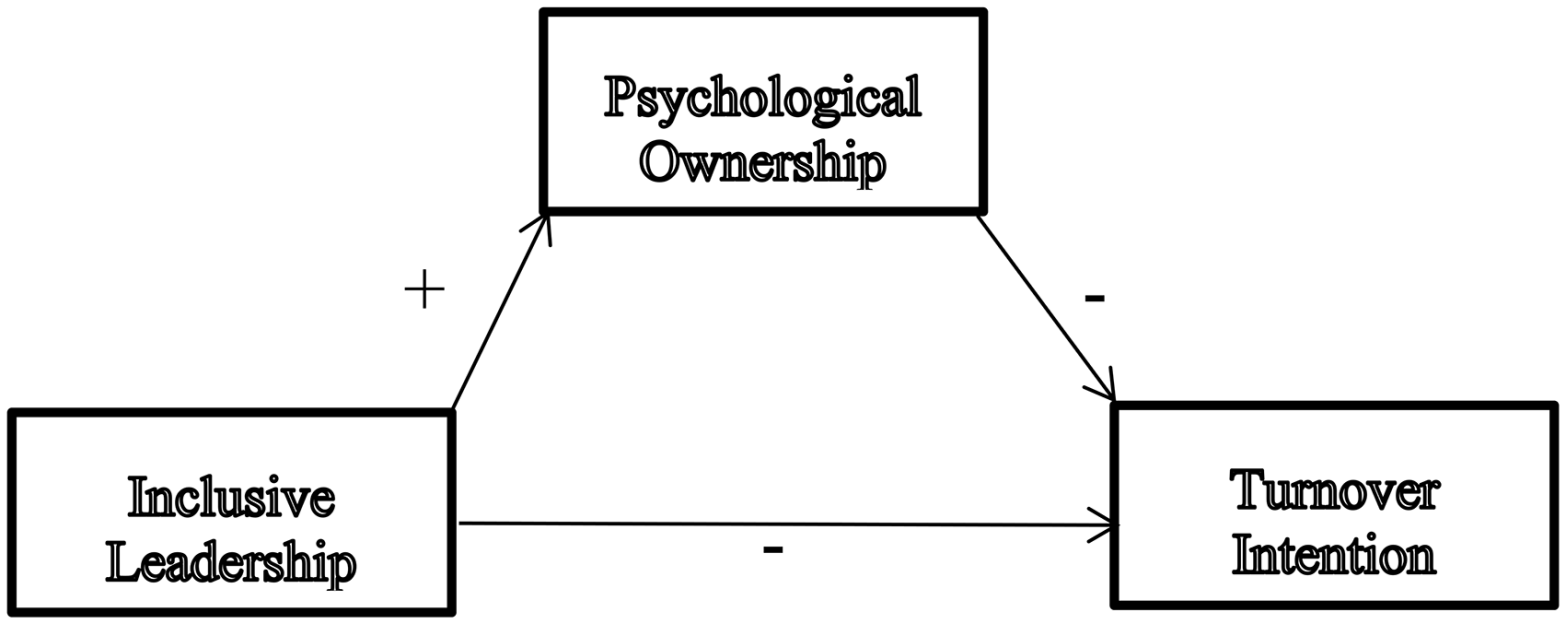
Mediating mechanism of psychological ownership.

A 2000-sampled maximum likelihood bootstrap was run with a 95 percent confidence level to examine the *p*-values using a two-tailed estimation. Table 6 shows independent variable inclusive leadership has a significant effect on the overall turnover tendency of nurses, *p* < 0.001; the direct effect path coefficient from inclusive leadership to psychological ownership is significant, *p* < 0.001, the direct effect path coefficient from psychological ownership to turnover intention is significant, *p* < 0.001, and the indirect effect path coefficient from inclusive leadership to turnover intention is significant, *p* < 0.001. In conclusion, psychological ownership acts as a partial intermediary in this study. The mechanism of its role as a mediating factor between inclusive leadership and turnover intention was shown as Figure 1. Path coefficients of mediating effects and standard regression equations were shown as Table 7.

**Table 6:** Results of two-tailed significance test of psychological ownership pathway.

	Step		Inclusive leadership	Psychological ownership
1	Total effect	Turnover intention	0.001	0.001
2	Direct effect	Psychological ownership	0.001	
		Turnover intention	0.001	0.001
3	Indirect effect	Turnover intention	0.001	

**Table 7:** Path coefficients of mediating effects and standard regression equations.

	Step	Path coefficients	Standard error	Standard regression equations
1	TI < - IL	−0.509	0.019	TI = −0.509*IL
2	PO < - IL	0.744	0.014	PO = 0.744*IL
	TI < - PO	−0.683	0.026	TI = −0.683*PO

^*^, multiplied by.

## 4. Discussion

This study aimed to identify how nurses in different hospital settings, age, marital status, educational background, work relationship, and experience feel about their leadership, their sense of psychological safety, and their tendency to quit. This study also examined the relationship between inclusive leadership, psychological ownership, and turnover intention, and verified the mediating role of psychological ownership between them. Numerous studies have explored the management of nurses under normal circumstances, but very few researchers have investigated the same during public health emergencies. This research gap has been filled by this study, which has revealed certain significant difference.

In terms of demographic characteristics, some results were not consistent with previous studies, which may be due to different cultural conditions, family structures, and gender roles of nurses in different countries and settings (Tolksdorf et al., 2022). In terms of age, the difference in inclusive leadership, psychological ownership, and turnover intention scores were statistically significant, which was not consistent with Tolksdorf’s research. She noted that age did not appear to be a factor in nurses’ turnover intentions, as was the case during the SARS pandemic from 2002 to 2004. However, in this study, nurses with higher age had higher scores for inclusive leadership and psychological ownership, and lower turnover intention. This may be because older nurses have more life experience, deeper social awareness, and are better at accommodating different perspectives, especially during a pandemic. Therefore, they can feel the inclusiveness of leadership and experience greater psychological safety. Most older nurses have a higher sense of belonging to their organization because of family obligations or a greater sense of responsibility. Additionally, they seek long-term, stable jobs, and therefore, quitting is not a fruitful option for them.

These results and interpretations about age also held true for nurses grouped by years of working. In this study, nurses with 1–5 years of working experience have a higher turnover intention; previous studies have reported that nurses with 1–3 years of working experience have an even higher turnover intention (Chang and Cho, 2022). These young nurses can quickly become stressed, overwhelmed, and discouraged in challenging environments. This study also found that nurses with less than 1 year of working experience had the lowest turnover intention, which was inconsistent with the results of Zhang’s study (Zhang et al., 2017). This may be due to the fact that hospital managers in China have adopted stronger positive policies for new nurses after realizing the shortage of nurses and high turnover rate of new nurses. For example, in the requirement of hospital evaluation standard, while evaluating the nurse’s performance, Grade 3 hospitals need to focus more on their actual labor, less on the working years and job title; therefore, young nurses, straight out of college, usually undergo more training, perform night shifts, handle greater workload compared to experienced nurses, and consequently, draw a higher salary, which can reduce their turnover intention. Additionally, the scores for inclusive leadership and psychological ownership perceived by nurses who had worked for less than 1 year were also the highest among the group of working years, which further explained the negative effect of inclusive leadership and psychological ownership on nurses’ turnover intention.

In China, co-chief superintendent nurses and above possessed significant professional experience, and had an average age of 36 years. They had higher scores of inclusive leadership and psychological ownership, and lower turnover intention compared to those with titles of nurse, senior nurse, and supervisor nurse.

Head nurses had the highest scores for inclusive leadership and psychological ownership, and lowest turnover intention among clinical nurses, group leaders, head nurses, and directors or the deputy directors of nursing department–this applies to China very well. In Chinese hospitals, head nurses not only undertake the tasks assigned by superior leaders, but also manage nurses in the ward. This makes the head nurse most personally aware of the management of the leadership relationship and they possess the maximum experience which contributes to their high sense of psychological safety. Additionally, for head nurses and above, their sense of personal achievement was also acquired at work; personal achievement is a predictor of nurses’ turnover intention (Karimi et al., 2022). When a person’s social status, personal income, and sense of achievement is acquired at work, they have a strong sense of belonging and identity toward the organization, higher psychological ownership, and lower turnover intention. Hence, the unique advantages of their position make it nearly impossible for them to leave.

Official established personnel labor relations could make nurses better equipped to cope with job instability in the face of a global pandemic. This may be because official established personnel labor relations provide higher after-tax income. Therefore, the results show that nurses with official established personnel labor relations and higher after-tax income level showed higher psychological ownership and lower turnover intention. Salary has been considered a significant factor affecting nurses’ turnover intention, which is consistent with previous studies (Widodo et al., 2021).

Unmarried nurses had higher turnover intention than married nurses, which may be caused by the differences in problems and responsibilities they face. When unmarried people leave, they often need to consider salary, personal development, and interests, and not worry about family or geographical issues. Married people, burdened with family responsibilities, need to consider more issues, such as future job prospects, the job of their spouse, the education of their children, etc. Therefore, this study shows that married nurses and those with two or more children had higher scores for inclusive leadership and psychological ownership, and lower turnover intention. Furthermore, the good health of family members can make nurses feel more psychologically safe during a pandemic.

Fewer night shifts and shorter working hours will also make nurses feel greater inclusive leadership relationship, more psychological security, and higher comfort at work, resulting in a lower intention to quit. Especially during a pandemic, reduction in working hours and intensity can relieve nurses’ stress.

How well nurses get along with colleagues and immediate leaders, support for the current job from family, and whether they work in their own city also significantly impact the performance of nurses. Getting along well with colleagues and immediate leaders make nurses have a good impression of work environment and feel that their leaders are congenial. Support from family and familiarity with the geography of the city improves their confidence at work, thereby reducing the probability of leaving the job.

Grade of hospital and work department could not adequately explain the different feelings of nurses toward inclusive leadership, psychological ownership, and turnover intention in this study. Group of professional character of hospital, designated hospitals for COVID-19 patients, experience of received COVID-19 patients, gender, and educational background showed little statistically significant difference.

The empirical analysis results for this study prove that inclusive leadership negatively predicts nurses’ turnover intention, inclusive leadership positively predicts their psychological ownership, and nurses’ psychological ownership negatively predicts their turnover intention. Nurses’ psychological ownership plays a partial mediating role between inclusive leadership and their turnover intention. This is consistent with the results of previous studies on employees in enterprises (Du et al., 2019). This study enriches the research results on the influence of inclusive leadership on employee turnover intention and the mediating effect of psychological ownership under different population characteristics and cultural backgrounds.

This study verified through empirical analysis that inclusive leadership in hospitals will have a negative impact on nurses’ turnover intention. In other words, the higher the level of inclusive leadership in a hospital, the lower the turnover intention of nurses, which is consistent with existing research. According to the leader-member exchange theory, the open, effective, and approachable style of inclusive leadership makes the relationship between leaders and nurses more harmonious. Inclusive leadership drives leaders to listen and pay attention to the needs of nurses and accept their individual characteristics. This facilitates the leader’s respect and trust for nurses, and based on their characteristics and strengths, leaders might provide the nurses resources and opportunities to help them achieve their goals and ideals. Nurses will be disinclined to quit under such working conditions and will develop a sense of identity with their hospitals or departments.

This study also verified through empirical analysis that inclusive leadership in hospitals will have a positive impact on nurses’ psychological ownership, which is a psychological state denoted by an individual’s emotions and cognition toward their organization. The psychological ownership toward the organization is mainly due to the organization’s ability to meet the three basic needs of the individual: the sense of belonging, self-efficacy, and self-identity. Inclusive leaders respect and understand the individual differences of employees, treat them fairly, encourage them to participate in the management and decision-making process of the organization, recognize their contributions to the organization, pay attention to communication with them, provide timely feedback, tolerate mistakes in principle, and assume active responsibility. This enhances employees’ sense of belonging to the organization and improves their self-efficacy and sense of identity with the organization (Sharif et al., 2021).

This study showed nurses’ psychological ownership negatively predicts their turnover intention. In other words, the higher the level of psychological ownership of nurses, the lower the turnover intention. Nurses with higher psychological ownership will identify more with their work and department, and have more positive emotions and a higher sense of responsibility for their work. Consequently, they can consciously improve personal work efficiency to provide higher quality nursing services to patients, and control negative intentions such as quitting.

This study constructed a structural equation model of inclusive leadership, psychological ownership, and turnover intention, and found the mediating role of psychological ownership between inclusive leadership and turnover intention. Hospital leaders adapt inclusive leadership management mode, treat nurses fairly, provide them with opportunities for self-development, and pay attention to communication with them. This results in a direct emotional exchange between leaders and nurses, because of which nurses feel good about their working environment and their turnover intention decreases. It also increases their level of psychological ownership by increasing their sense of belonging, self-efficacy, and self-identity, which further lowers their probability of quitting.

In the context of the COVID-19 pandemic, the management of nurses has become a serious problem. How to make nurses maintain their recognition of the organization, have good psychological ownership, and not easily entertain the idea of quitting are a few of the challenges currently faced by hospital managers. The empirical findings of this study show a few differences in nurses’ perceived inclusive leadership, psychological ownership, and turnover intention under different demographic variables. Therefore, managers should formulate targeted programs based on the characteristics of nurses and hospitals. Increasing the proportion of nurses with official institution personnel establishment, reducing the number of night shifts and weekly working hours, increasing salary, and improving the working environment between leaders and nurses are all feasible measures to improve inclusive leadership, enhance nurses’ identification with the organization, and reduce the level of turnover intention. Based on the negative predictive and moderating effects of inclusive leadership and psychological ownership on turnover intention, hospital leaders should strengthen the implementation of inclusive leadership training courses for managers at all levels, introduce its core content to the front-line nurses, and publicize the crucial role of inclusive leadership in the context of COVID-19.

From another perspective, managers should pay more attention to nurses’ emotional distress, based on the mediating mechanism of psychological ownership revealed in this study. In Busch et al. (2021)‘s systematic review and meta-analysis of the psychological burden of frontline healthcare workers in the last two decades of epidemics and pandemics, it revealed a core reason of global psychological distress is the perceived lack of control of one’s own professional and personal life. Therefore, clear and transparent crisis communication, updated information, psychological support and flexible work schedules should be provided by managers adapting inclusive leadership style at the organizational and structural level. As for individual level, healthcare workers could seek for peer support programs and strengthen communication with peers, in order to get easy and timely access to psychological first aid and emotional support. Striving to improve professional skills is also a way for healthcare providers to reduce psychological distress, because it can increase the ability to cope with unexpected events.

## 5. Limitations and future research

This study is only relevant to nurses during the period of COVID-19 pandemic prevention; therefore, it may not be applicable to other groups and extra attention should be paid when applying the results to other groups. Similarly, our results may be subject to some bias when it is not in the context of COVID-19. More than 98.5% of the sample data in this study was derived from nurses in hospitals in the Guangdong Province; future studies should consider collecting an even proportion of samples across provinces. 97.3% of the sample was composed by female nurses, which may also cause some bias. An online questionnaire was used in this study. It is possible that the lower completion rate and selective participation that are common with web-based questionnaires could have influenced the point estimates of the dependent variable in this study. In the future, a multi-center data source study could be conducted to extend the representativeness of the findings. Additionally, researchers can consider conducting longitudinal research on inclusive leadership, for example, to study the effects of inclusive leadership on turnover intention of nurses during and after the COVID-19 pandemic.

## 6. Conclusion

Under the current situation of COVID-19 prevention, many front-line nurses suffer from psychological anxiety, burnout, turnover intention, and even suicidal thoughts. If a mechanism cannot be found soon to counteract these negative conditions, it may ultimately lead to personal psychological distress for nurses and the collapse of the healthcare system. This study showed that inclusive leadership had a significant effect on increasing nurses’ psychological ownership and reducing their turnover intention during the COVID-19 pandemic. Inclusive leadership can improve nurses’ psychological ownership level and reduce their turnover intention by treating them fairly, providing them with opportunities for self-development, paying attention to communication with them, and increasing their sense of belonging, self-efficacy, and self-identity.

## Data availability statement

The original contributions presented in the study are included in the article/supplementary material, further inquiries can be directed to the corresponding author.

## Ethics statement

Before commencement of this research, ethical approval (No. KY-2022-225) was obtained from the Ethics Committee of the First Affiliated Hospital of Jinan University.

## Author contributions

DZ contributed to conception of the research study, carried out the research, and played a role in collection of data. BW contributed to the analysis and interpretation of data and working out of the manuscript. WC played a significant supervisory role and contributed to the final manuscript. All authors contributed to the article and approved the submitted version.

## Conflict of interest

The authors declare that the research was conducted in the absence of any commercial or financial relationships that could be construed as a potential conflict of interest.

## Publisher’s note

All claims expressed in this article are solely those of the authors and do not necessarily represent those of their affiliated organizations, or those of the publisher, the editors and the reviewers. Any product that may be evaluated in this article, or claim that may be made by its manufacturer, is not guaranteed or endorsed by the publisher.
